# COVID-19 disruption to cervical cancer screening in England

**DOI:** 10.1177/09691413221090892

**Published:** 2022-09

**Authors:** Alejandra Castanon, Matejka Rebolj, Francesca Pesola, Philippa Pearmain, Ruth Stubbs

**Affiliations:** 1 School of Cancer & Pharmaceutical Sciences, 4616 King’s College London, London, UK; 2 Screening Quality Assurance Service, NHS England and NHS Improvement, London, UK; 3 Public Health Commissioning and Operations, NHS England and NHS Improvement, London, UK

**Keywords:** cervical cancer screening, COVID-19, excess cervical cancer, screening delays

## Abstract

**Introduction:**

In England, routine invitations for cervical screening were reduced between April 2020 and June 2020 due to the COVID-19 pandemic. We quantify the impact of COVID-19 disruptions on attendance and excess diagnoses of cervical cancer (CC).

**Methods:**

Using Public Health England CC screening data on laboratory samples received in 2018 as a baseline we quantify the reduction in screening attendances due to the COVID-19 pandemic between April 2020 and March 2021 for women aged 25–64. We model the impact on excess CC diagnoses assuming once invitations resume 87.5% of women attend within 12 months and 12.5% delay screening for 3 or 5 years (depending on age).

**Results:**

The number of samples received at laboratories was 91% lower than expected during April, 85% during May and 43% during June 2020 compared to the same period in 2018. Although on average laboratories received 12.6% more samples between August 2020 and April 2021 than over the same months in 2018, by April 2021 there was a short fall of 200,949 samples (6.4% fewer than in 2018). An excess of 41 CC (4.0 per 100,000 women with a maximum screening delay of 12 months) are predicted to occur among the estimated 1,024,794 women attending this screening round with a delay. An excess of 60 CC (41.0 per 100,000 women) are predicted to occur among the estimated 146,391 women who do not attend this screening round.

**Conclusion:**

Prompt restoration of cervical screening services limited the impact on excess CC diagnoses. However, in 2020 a 6.4% shortfall of screening samples was observed. Every effort should be made to reassure these women that services are open and safe to attend.

## Introduction

Disruptions to screening due to the COVID-19 pandemic have been observed the world over.^1–3^ The UK Office for National Statistics estimated that the first wave of COVID-19 started in March 2020 and ended by the end of May 2020.^4^ Whilst, in England, cervical screening did not officially stop due to the pandemic, the volume of screening invitation letters was reduced from early April to June 2020.^5^ During June 2020, invitations were restricted to women on early recall so that they could be caught up first, as they have a higher risk of underlying lesions since they have previously tested positive for high-risk human papillomavirus (hr-HPV). England experienced a second wave of the pandemic (estimated to have started in September 2020 and ended in April 2021).^4^ During that wave, screening services remained open but women's reluctance to attend screening may still have affected attendance rates.

Cervical screening in England is offered to women aged 25–49 at three-yearly intervals and to those aged 50–64 at five-yearly intervals. All women receive personal invitation letters once they are due for screening or early recall testing, with reminders sent 3 months later. Screening is mostly carried out in general practise by specially trained nurses and was done using liquid-based cytology until December 2019, when the national roll out of hr-HPV primary testing was completed. The aim is to identify women who are at increased risk due to having a hr-HPV and abnormal cells in the cervix, so that their abnormalities can be treated before cancer develops. The aim of screening, therefore, is to reduce the numbers of women diagnosed with cervical cancer (CC) and those who die from it, for which hr-HPV testing is more effective than cytology.^6^ In the coming decades, these numbers are likely going to diminish also due to vaccination against most high-risk genotypes.^7^ Vaccination against HPV (bi-valent vaccine protecting from genotypes 16 and 18) was introduced in England in 2008 for girls aged 12–13 years (born from 1st of September 1995 onward) and, in 2008–2010, to a catch-up cohort aged 14–18 years (born from 1st of September 1990 to 31st of August 1995). Coverage among girls aged 12–13 years has remained around 86%.^8^

We have previously estimated the effect of the COVID-19 disruption on CC incidence in England under a range of assumptions about the length of the disruption and the screening programme's approach to compensate for the lost opportunity to be screened.^9^ We now have better information on the effect of the disruption on the numbers of cervical samples processed in screening laboratories, and how those numbers changed on a monthly basis. Hence, we aimed to quantify the impact of the pandemic disruption on the incidence of CC among routine attenders to screening in England with greater precision than has so far been possible.

## Methods

**Figure 1.**
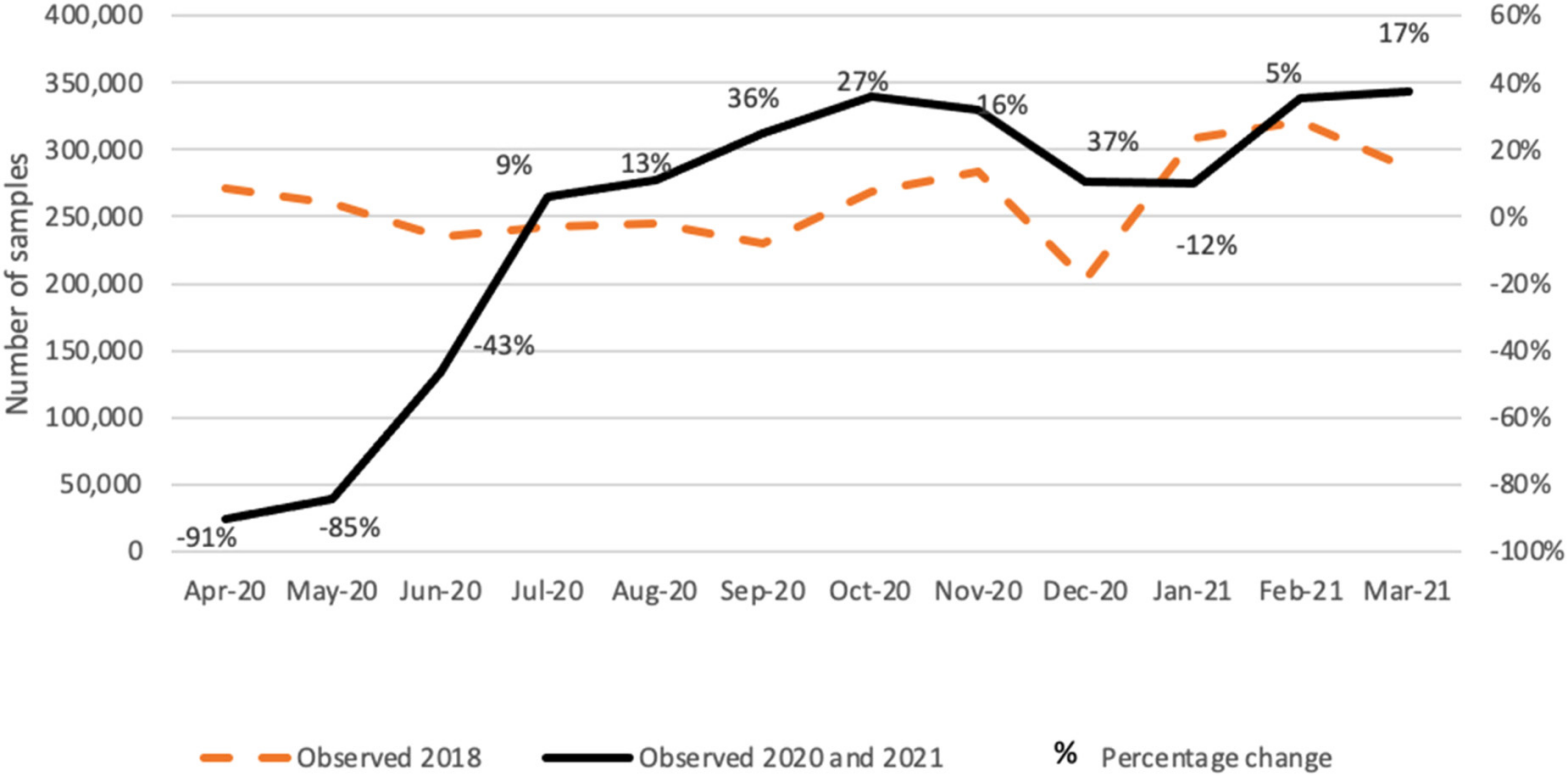
Number of samples received at laboratories in England and percentage change between samples received between January and December 2018 and those received between April 2020 and March 2021.

We used data from Public Health England (PHE) routine statistics reporting the numbers of laboratory samples received and booked onto the laboratory data system (Figure 1). This source, which includes all cervical samples processed for either hr-HPV testing or cytology, served as a proxy for the number of women who attended screening between January 2018 and March 2021.

Year 2018 was used as the baseline representing a “normal” screening year during which women could attend screening without delays. Year 2019 was not considered for a baseline. In that year, PHE ran a successful public campaign promoting screening uptake.^10^ The change from cytology to hr-HPV-based primary screening would not have changed the number of women invited to screening in 2020 and 2021 as the next invitation date is set based on the previous test result which would have been carried out using liquid-based cytology.

We estimated the impact of COVID-19 disruptions on screening participation among women aged 25–64 who were due to be sent an invitation between April 2020 and December 2021, and on the excess CC incidence as a consequence of missed screening appointments. For April to December 2021, laboratory sample data have not yet been made available. As the data indicated that the monthly volumes of samples returned close to the normal levels, we assumed that the numbers of women attending screening each month during this period was equal to the numbers reported during 2018.

To model the impact of an increase in samples received from April to December 2021, observed laboratory samples in 2018 were increased by 5% each month and this figure was taken as the total samples processed each month between April and December 2021. In this analysis the additional ‘capacity’ allows more women to be screened on time reducing the total number with delays to screening of up to 12 months.

The difference between the numbers of women expected to attend for screening each month (i.e. baseline estimates from January to December 2018) and the numbers attending each month during April 2020 to December 2021 was assumed to be the number of women who had had their screening delayed (if it was a positive number) or the additional number of women that had been tested in that month i.e., women who had been caught up with screening after the delay (if it was a negative number). Note that women could have their screening delayed both between April and June 2020 and thereafter either due to lack of appointments or because they continued to be hesitant to attend. We were agnostic to the reasons for the delay.

## Attendance at screening following an invitation (or a reminder) letter

A survey of women's attitudes to screening carried out by Jo's CC Trust during the first months of the pandemic found that approximately 10–15% of women were unlikely to attend screening because of fears related to COVID-19.^11^ Hence, we assumed that 12.5% of women will delay screening until their next invitation (3 or 5 years later depending on age).

We accounted for the usual delays in attending for screening after receiving an invitation. The distribution of attendance within the first 12 months after an invitation was estimated from the English HPV primary screening pilot.^12^Supplementary Table 1 summarises assumed attendance distribution.

In each month from July 2020 onwards, priority for attendance in the model was given to those women who were estimated to have experienced a delay due to COVID-19 disruptions, so that their delays would be minimised. If, in a given month, the number of such women was lower than the observed number of samples processed in English screening laboratories, we assumed that the remaining screening samples processed in that month were from women who were due to attend following a regular schedule, i.e. without a delay. Women on a regular screening schedule who could not be screened in that month were assumed to attend following the distribution described in Table S1. This process was repeated until December 2021 which means that the last individual affected by a delay will either have attended by December 2022 or will have delayed screening for the full 3- (or 5)-year screening round, depending on age.

## Age distribution among women attending screening

The monthly numbers of laboratory samples reported to PHE were not broken down by women's age. Since the likelihood of developing high-grade cervical intraepithelial neoplasia (CIN) and CC is age dependent, we used proportions of women attending screening in five-year age groups from age 25 to 64 from the NHS Cervical Screening Programme statistics for financial year 2018/19.^13^ These statistics suggested that out of the total number of women tested in that year, 16.8% were aged 25–29, 15.4% aged 30–34, 15.2% aged 35–39, 13.4% aged 40–44, 13.9% aged 45–49, 10.8% aged 50–54, 8.0% aged 55–59 and 6.5% aged 60–64.

First we estimated the total women delayed by calendar month (women are delayed in the same way regardless of age) and then applied the age distribution.

## Population with cervical intraepithelial neoplasia

To estimate the number of screened women with a high-grade CIN detectable through hr-HPV screening, data from the first round within the English HPV primary screening pilot was used, when by far the majority of women were unvaccinated.^12^ The pilot reported that 6.6% women aged 25–29, 1.6% aged 30–59 and 0.5% aged 60–64 had a CIN grade 2 or worse detected following hr-HPV primary screening, either at baseline or at one of two early recalls. By far most women in England who were due for screening in 2020 and 2021 would have attended hr-HPV-based screening for the first time.

The numbers of women with a high-grade CIN lesion detected following a delay to screening were estimated by multiplying the above proportions and the estimated numbers of women with a screening delay. The proportion of high-grade CIN that would have progressed to CC, depending on the number of months by which screening was delayed, was based on progression estimates from the Landy et al.^14^ modelling study. In that study, parameter sets were chosen to be consistent with the literature, otherwise they were rejected. Cumulative transition probabilities (following an exponential distribution)^15^ from 1 month up to 12 months, and separately for 36 months (for women aged 25–49 years) or 60 months (for women aged 50–64 years) for those missing the whole screening round, are presented in Table S2.

Estimates of the numbers of women in whom delays to screening led to progression of high-grade CIN to CC were adjusted for vaccination coverage in women aged 25–29 and 30–34. The proportion of the population by age at vaccination and calendar year who are assumed to have been protected by vaccination^16^ is presented in Table S3. Adjustments were made separately for those attending between June and August 2020 and for those attending from September 2021 because the proportions of vaccinated women were different.

The odds ratio of being diagnosed with a CIN grade 3 or worse by age at vaccination as reported for Scotland^17^ were used to adjust the proportion of high-grade CIN per 100,000 women screened to better reflect the risk in vaccinated women. Compared to unvaccinated women, odds of high-grade CIN were the lowest (OR = 0.14) among those vaccinated at ages 12 or 13 years. Thereafter, the odds ratio increased to 0.18 in those vaccinated age 14 years, 0.29 at age 15 years, 0.27 at age 16 years, 0.55 at age 17 years and 0.85 at age 18 years.

## Results

The number of samples received at laboratories in England was 91% lower than expected during April 2020, 85% during May 2020, and 43% during June 2020 (Figure 1). Henceforth we refer to the period between April and June 2020 as the acute phase of the pandemic, and to the period from July 2020 onwards as the recovery phase.

Although on average laboratories received 12.6% more samples between August 2020 and April 2021 than over the same months in 2018, by April 2021 there was still a shortfall of 200,949 samples (6.4% fewer than expected before the pandemic). This number represents on average about three weeks of screening under pre-pandemic circumstances.

During the acute phase of the COVID-19 pandemic (April to June 2020), 467,687 women were estimated to have experienced a disruption to screening. Of these women, 58,461 (12.5%) would not attend screening during the current round and instead return 3 or 5 years from now (i.e. between 2023 and 2025). We estimated that all women who had their screening delayed during the acute phase and who would still attend, could do so by June 2021.

An additional 703,440 women were estimated to have their screening delayed during the recovery phase (from July 2020 to December 2021). Of these, 87,930 would not attend until the next age-appropriate screening round.

**Figure 2.**
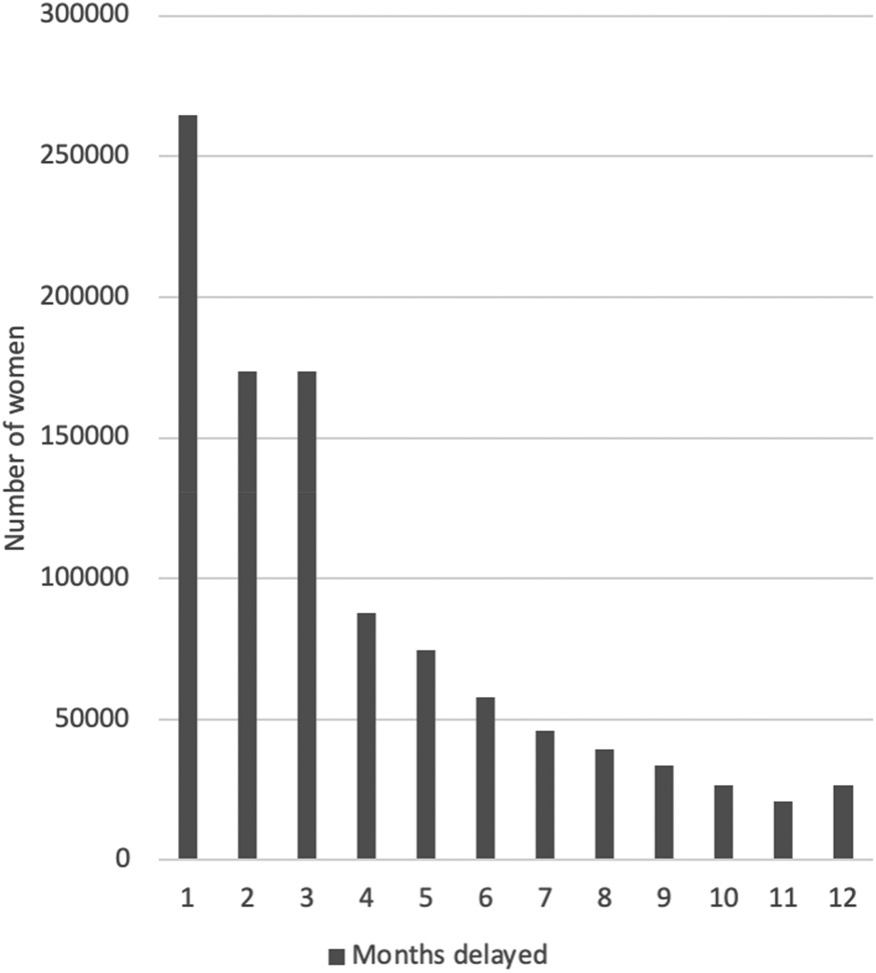
Number of women whose screening was delayed by up to 12 months, by number of months delay.

Of the estimated 1,024,736 women who experienced a screening delay of up to 12 months, the majority (52.3%) would be delayed up to 3 months, with an additional 18.8% having a delay of between 4 and 6 months (Figure 2). Among women with a delay of up to 12 months, an excess of 41 cases of CC (4.0 per 100,000 women with a maximum screening delay of 12 months) are expected (Table 1). Women in their 40s have the greatest burden of excess cancer diagnoses.

**Table 1.** Estimated excess cervical cancer incidence for women who attend with a maximum delay of 12 months, by age at invitation to screening.

Age group at invitation	Population attending with a delay	Total with high-grade CIN	Total excess cancers	Rate per 100,000 delayed
**24–29**	172,173	6188	5	2.8
**30–34**	157,887	2417	4	2.5
**35–39**	155,859	2494	6	3.6
**40–44**	137,041	2193	9	6.7
**45–49**	142,721	2284	10	6.7
**50–54**	110,465	552	3	2.9
**55–59**	82,230	411	2	2.9
**60–64**	66,362	332	2	3.5
**Total**	1,024,736	16,870	41	4.0

CIN: cervical intraepithelial neoplasia.

A total of 60 excess cancers were estimated to occur among the 146,391 women who delay their screening until the next round on account of the COVID-19 pandemic. This is equivalent to a rate of 41.0 per 100,000 women delaying screening until the next round (Table 2).

**Table 2.** Estimated excess cervical cancer incidence for women missing a whole screening round, by age at invitation to screening.

Age group at invitation	Population attending with a delay	Total with high-grade CIN	Total excess cancers	Rate per 100,000 delayed
**24–29**	24,596	893	6	26.0
**30–34**	22,555	347	5	22.9
**35–39**	22,266	356	7	33.2
**40–44**	19,577	313	12	61.2
**45–49**	20,389	326	12	61.2
**50–54**	15,781	79	7	42.0
**55–59**	11,747	59	5	43.0
**60–64**	9480	47	5	52.1
**Total**	146,391	2420	60	41.0

CIN: cervical intraepithelial neoplasia.

Assuming that from April 2021 onwards there was no significant increase in the number of screening samples processed in English laboratories as compared with the numbers observed prior to COVID-19 disruption, women would likely continue to experience delays into 2023 (outcomes for these women have not been modelled). However, if the number of samples from April 2021 onwards could be increased by 5% above the levels observed in 2018, then the backlog accrued during the recovery period could be cleared by the end of June 2021 and 9 excess cancers prevented (Table 3).

**Table 3.** Outcome for women who attend with a maximum delay of 12 months, by age at invitation to screening, assuming attendance increased 5% above expected every month from April to December 2021.

Age group at invitation	Population attending with a delay	Total with high-grade CIN	Total excess cancers	Rate per 100,000 delayed
**24–29**	128,091	4843	4	3.0
**30–34**	117,463	1809	3	2.6
**35–39**	115,954	1855	4	3.8
**40–44**	101,954	1631	7	7.0
**45–49**	106,179	1699	7	7.0
**50–54**	82,182	411	2	3.0
**55–59**	61,176	306	2	3.0
**60–64**	49,371	247	2	3.7
**Total**	762,369	12,801	32	4.2

CIN: cervical intraepithelial neoplasia.

## Discussion

Following a three-month disruption to cervical screening in England, 101 excess cases of CC were estimated to be diagnosed. For comparison, an average of 2626 cases were diagnosed per year in England in 2016–2018.^18^ Consistently with a previous analysis,^9^ women who delay their screening for an entire 3- or 5-year round (depending on age) would be affected the most However, that study assumed that all women would experience the same delays and estimated an excess of 315 cervical cancers following a three-month disruption with no increase in capacity.^9^ This is three times as many as estimated in the current study. It would appear that the English programme's ability to process an average of 20% more samples between August and December 2020 compared to pre-pandemic levels, combined with a rapid restoration of services, has contributed to reduce the excess numbers of CC diagnoses.

We now know that English laboratories have been able to restore cervical screening services above pre-pandemic levels. Data on samples processed after April 2021 were not available. If the increase in screening attendance and laboratory testing volumes could be maintained at least 5% above the pre-pandemic levels for the rest of 2021, COVID-19-related backlogs may have been cleared as soon as June 2021, further limiting the impact on excess CC diagnoses.

This study has several limitations. The data on laboratory samples used for this study are not equivalent to the number of women screened, and will include a small proportion of samples from the same women, e.g. due to repeated sampling after an inadequate sample. Further, it included some women on early recall and those attending for an hr-HPV test following CIN treatment. Since no details on the screening history profile of those attending screening were available, we assumed that women who attend screening in the post-pandemic recovery period had similar attendance patterns as observed before the pandemic, and an average risk of high-grade CIN and CC. Consistent with survey data,^11^ we assumed that 12.5% of previous regular attenders would not participate in this screening round. If fewer women delayed their screening for three or more years, fewer excess cancers would be observed. However, this would have increased the proportion of women delayed by up to 12 months during the recovery phase of the pandemic impacting backlogs to screening in 2021. It is worth noting that for the 12.5% of women who do not participate in this screening round we modelled the proportion who would develop cancer over a 3 or 5-year period but remain agnostic to whether these cancers were screen detected or not. Among women aged 60–64 one would expect some cancers to be diagnosed following presentation with symptoms, since they would not all be invited again for screening (depending on when they were last screened).

NHS England and NHS Improvement (NHSEI) issued guidance^19^ in December 2020 urging providers to prioritise screening service maintenance without diverting screening staff towards other services. This guidance appears to have helped maintain screening services at pre-pandemic levels during January and February 2021, the second wave of the pandemic.

We have not modelled the impact of delays to those referred to colposcopy for further investigation. A joint communication from the Royal College of Obstetricians and Gynaecologists and the British Society for Colposcopy and Cervical Pathology in March 2021 indicated that colposcopy services were limited during the acute phase of the pandemic and that it had taken several months for them to recover, with many clinics, at that time, still unable to offer the same number of appointments as before the pandemic, due to local guidance on social distancing, increased cleaning, etc.^20^ The authors are aware of a small number of clinics with delays to colposcopy services for some groups of patients where the priority for appointments was given to those with a higher risk of cervical abnormality. However, this is not universal across England. In addition, 2020 saw the rollout of hr-HPV primary testing and a surge in colposcopy referrals was expected from 2021 onwards. If colposcopy capacity is in fact constrained, then this may have knock-on effects which may not be apparent until well into 2021 (data not available yet). The approach to prioritise patients with the highest likelihood of significant cervical abnormality and cancer will mitigate this potential impact.

Other studies^21,22^ that assessed the potential impact of COVID-19 disruptions to cervical screening using microsimulation models have also shown relatively small effects of the disruption on CC incidence and the clear advantage of a rapid catch-up on minimising excess cancers.

## Conclusion

The prompt restoration of cervical screening services appears to have limited the impact of the COVID-19 disruption on excess cancer diagnoses. However, a 6.4% shortfall in screening samples was observed between March 2020 and April 2021. Given the higher excess risk among women who delay their screening for 3 years or more, every effort should be made to promote the programme and reassure these women that services are open and safe to attend. This is particularly important while the COVID-19 pandemic continues, to avoid further increasing the number of women who delay their screening.

## Supplemental Material

sj-docx-1-msc-10.1177_09691413221090892 - Supplemental material for COVID-19 disruption to cervical cancer screening in England Click here for additional data file. Supplemental material, sj-docx-1-msc-10.1177_09691413221090892 for COVID-19 disruption to cervical cancer screening in England by Alejandra Castanon, Matejka Rebolj, Francesca Pesola, Philippa Pearmain and Ruth Stubbs in Journal of Medical Screening
