# Supplementary material for: COVID-19 disruption to cervical cancer screening in England
Source: J Med Screen. 2022 Sep;29(3):203–8. doi: 10.1177/09691413221090892 (PMC9381684; doi:10.1177/09691413221090892)
Supplement: sj-docx-1-msc-10.1177_09691413221090892 - Supplemental material for COVID-19 disruption to cervical cancer screening in England [file sj-docx-1-msc-10.1177_09691413221090892.docx]

Supplementary Tables

|  | **Table S1. Distribution of attendance at screening within 12 months of an invitation.** | | | | | | | | | | | | | | | | |
| --- | --- | --- | --- | --- | --- | --- | --- | --- | --- | --- | --- | --- | --- | --- | --- | --- | --- |
|  | | Proportion of women who will attend in each month after invitation | | | | | | | | | | | | |  | |  |
| **Months since invitation** | | **1mth**^1^ | **2mth** | **3mth** | **4mth** | **5mth** | **6mth** | **7mth** | **8mth** | **9mth** | **10mth** | **11mth** | **12mth** |  | |  |  |
| Attendance distribution^2^ | | 43.0% | 14.7% | 8.5% | 6.4% | 7.1% | 4.9% | 3.4% | 3.6% | 3.1% | 2.1% | 1.6% | 1.5% |  | |  |  |
|  | |  |  |  |  |  |  |  |  |  |  |  |  |  | |  |  |

^1^ Includes women who would normally attend prior to an invitation or on the month of invitation (i.e. on time)

^2^ Among women who participated in the English HPV screening pilot’s first round in 2013 at the age of 24-59 (N=167,981), and were returned to age-appropriate routine recall following negative results in either liquid-based cytology or HPV screening, we established whether they had a new screening sample reported to the pilot's central database within the next 3 or 5 years (depending on their age) or within the following 12 months. If such a sample was registered, we determined the number of months between the first and the second screening samples. Women were considered to have been screened "on time" if their second screening sample was taken exactly 3 years (36.0-36.99 months) after the first one at age 24.5-49, and exactly 5 years (60.0-60.99 months) after the first one at age 50-59. For other women, the timeliness of their second samples was determined accordingly.

Table S2. Monthly transition probabilities from high-grade cervical intraepithelial neoplasia to cervical cancer, by age.

|  | Transition from high-grade CIN^1^ to cervical cancer | | | | | |
| --- | --- | --- | --- | --- | --- | --- |
| Age group | 25-29 years | 30-34 years | 35-39 years | 40-49 years | 50-59 years | 60-64 years |
| Months of screening delay |  |  |  |  |  |  |
| 1mth | 0.02% | 0.04% | 0.06% | 0.11% | 0.15% | 0.18% |
| 2mth | 0.04% | 0.08% | 0.12% | 0.22% | 0.30% | 0.37% |
| 3mth | 0.06% | 0.12% | 0.17% | 0.32% | 0.45% | 0.55% |
| 4mth | 0.08% | 0.17% | 0.23% | 0.43% | 0.60% | 0.73% |
| 5mth | 0.10% | 0.21% | 0.29% | 0.54% | 0.75% | 0.91% |
| 6mth | 0.12% | 0.25% | 0.35% | 0.65% | 0.90% | 1.10% |
| 7mth | 0.14% | 0.29% | 0.41% | 0.76% | 1.04% | 1.28% |
| 8mth | 0.16% | 0.33% | 0.47% | 0.86% | 1.19% | 1.46% |
| 9mth | 0.18% | 0.37% | 0.52% | 0.97% | 1.34% | 1.64% |
| 10mth | 0.20% | 0.42% | 0.58% | 1.08% | 1.49% | 1.82% |
| 11mth | 0.22% | 0.46% | 0.64% | 1.18% | 1.64% | 2.00% |
| 12mth | 0.24% | 0.50% | 0.70% | 1.29% | 1.78% | 2.18% |
| 36mth | 0.72% | 1.49% | 2.08% | 3.82% | 5.26% | 6.39% |
| 60mth | 1.19% | 2.47% | 3.44% | 6.29% | 8.61% | 10.42% |
| ^1^ Cervical intraepithelial neoplasia (CIN) | | | | | | |

| Table S3. Proportion of the population protected against HPV^1^ 16/18 by age at vaccination and calendar year at fist screen. | | | | | | | | | | |
| --- | --- | --- | --- | --- | --- | --- | --- | --- | --- | --- |
| Vaccine age | Birth cohort | Coverage*  (three doses) | Protected against HPV  16/18 | Year enter screening | Proportion in the population aged 24-29yrs protected | | | Proportion in the population aged 30-34yrs protected | | |
|  |  |  |  |  | 2020 | 2021 | 2022 | 2020 | 2021 | 2022 |
| 12/13 | 01 Sept 95 – 31 Aug 97 | 80.9-84.4% | 100% | 2020-2022 | 17% | 34% | 51% | - | - | - |
| 14/15 | 01 Sept 94 – 31 Aug 95 | 75.7% | 95% | 2019 | 14% | 14% | 14% | - | - | - |
| 15/16 | 01 Sept 93 – 31 Aug 94 | 70.8% | 90% | 2018 | 13% | 13% | 13% | - | - | 13% |
| 16/17 | 01 Sept 92 – 31 Aug 93 | 48.1% | 85% | 2017 | 8% | 8% | - | - | 8% | 8% |
| 17/18 | 01 Sept 91 – 31 Aug 92 | 38.9% | 80% | 2016 | 6% | - | - | 6% | 6% | 6% |
| 17/18 | 01 Sept 90 – 31 Aug 91 | 47.4% | 80% | 2015 | - | - | - | 8% | 8% | 8% |

^1^ Human Papillomavirus (HPV)

*Coverage obtained from 2010/11 vaccine coverage figures for England.(16)
